# Human Neuromuscular Junction on a Chip: Impact of Amniotic Fluid Stem Cell Extracellular Vesicles on Muscle Atrophy and NMJ Integrity

**DOI:** 10.3390/ijms24054944

**Published:** 2023-03-03

**Authors:** Martina Gatti, Katarina Stoklund Dittlau, Francesca Beretti, Laura Yedigaryan, Manuela Zavatti, Pietro Cortelli, Carla Palumbo, Emma Bertucci, Ludo Van Den Bosch, Maurilio Sampaolesi, Tullia Maraldi

**Affiliations:** 1 Department of Biomedical, Metabolic and Neural Sciences, University of Modena and Reggio Emilia, 41125 Modena, Italy; 2 Department of Neurosciences, Experimental Neurology and Leuven Brain Institute, KU Leuven—University of Leuven, 3000 Leuven, Belgium; 3 VIB, Center for Brain & Disease Research, Laboratory of Neurobiology, 3000 Leuven, Belgium; 4 Department of Development and Regeneration, Stem Cell and Developmental Biology, KU Leuven—University of Leuven, 3000 Leuven, Belgium; 5 Department of Biomedical and Neuromotor Sciences (DIBINEM), University of Bologna, 40127 Bologna, Italy; 6 Department of Medical and Surgical Sciences for Mothers, Children and Adults, Azienda Ospedaliero Universitaria Policlinico, University of Modena and Reggio Emilia, 41124 Modena, Italy; 7 Histology and Medical Embryology Unit, Department of Anatomy, Histology, Forensic Medicine and Orthopedics, Sapienza University of Rome, 00185 Rome, Italy

**Keywords:** neuromuscular junction, amniotic fluid stem cells, extracellular vesicles, muscle atrophy, oxidative stress

## Abstract

Neuromuscular junctions (NMJs) are specialized synapses, crucial for the communication between spinal motor neurons (MNs) and skeletal muscle. NMJs become vulnerable in degenerative diseases, such as muscle atrophy, where the crosstalk between the different cell populations fails, and the regenerative ability of the entire tissue is hampered. How skeletal muscle sends retrograde signals to MNs through NMJs represents an intriguing field of research, and the role of oxidative stress and its sources remain poorly understood. Recent works demonstrate the myofiber regeneration potential of stem cells, including amniotic fluid stem cells (AFSC), and secreted extracellular vesicles (EVs) as cell-free therapy. To study NMJ perturbations during muscle atrophy, we generated an MN/myotube co-culture system through Xona^TM^ microfluidic devices, and muscle atrophy was induced in vitro by Dexamethasone (Dexa). After atrophy induction, we treated muscle and MN compartments with AFSC-derived EVs (AFSC-EVs) to investigate their regenerative and anti-oxidative potential in counteracting NMJ alterations. We found that the presence of EVs reduced morphological and functional in vitro defects induced by Dexa. Interestingly, oxidative stress, occurring in atrophic myotubes and thus involving neurites as well, was prevented by EV treatment. Here, we provided and validated a fluidically isolated system represented by microfluidic devices for studying human MN and myotube interactions in healthy and Dexa-induced atrophic conditions—allowing the isolation of subcellular compartments for region-specific analyses—and demonstrated the efficacy of AFSC-EVs in counteracting NMJ perturbations.

## 1. Introduction

Skeletal muscle is a very plastic tissue, but its regenerative potential is hampered during aging [1]. The loss of muscle mass and function associated with muscle-wasting conditions greatly affects the quality of life in elderly populations [2]. Muscle atrophy is characterized by an activation of proteolytic systems that leads to the elimination of contractile proteins and organelles, with loss of skeletal muscle mass, quality, and strength [1,3]. In addition to this, the loss of alpha motor neurons (MNs) and negative alterations of neuromuscular junctions (NMJs) play a key role in musculoskeletal impairment that occurs with aging [4,5]. NMJs are specialized regions where muscle and nerve can communicate—a fundamental connection to govern vital processes, such as breathing and voluntary movements [6]. In physiological conditions, after neuronal loss, denervated orphan muscle fibers, together with some other types of cells, such as terminal Schwann cells, produce chemotactic signals that stimulate the growth of new neurites and, consequently, their re-innervation. These compensatory strategies start failing with aging and the fibers that have not re-innervated become apoptotic, leading to a decline in muscle capabilities [7,8,9]. Moreover, the depletion of adult satellite cells (SCs), the characteristic muscle stem cell compartment, aggravates this dramatic context [10]. This loss in muscle integrity leads to alterations in NMJ morphology that becomes fragmented, and to functional changes in neuromuscular transmission [11]. This initial NMJ change is accompanied by an increase in inflammatory cytokine production and loss of trophic support with consequent neurodegeneration [12].

Furthermore, the age-associated increase in oxidative stress and mitochondrial dysfunction plays a crucial role in NMJ degeneration and muscle atrophy. This oxygen metabolism defect, associated with the reduction in mitochondrial energy production and increase in intracellular calcium, intensifies the pre-synaptic decline and reduces the release of synaptic vesicles. The increase in reactive oxygen species (ROS)—due to mitochondrial dysfunction—in both muscle and neural tissues leads to an accumulation of damaged cell components with alteration in their communication [6,13]. Nevertheless, in this fundamental crosstalk, it has not yet been clarified whether NMJ alteration precedes or follows muscle decline, nor what role oxidative stress components play under such circumstances. Based on these considerations, combining neuro-muscular protection, anti-inflammatory, and antioxidant capabilities may be a promising way to counteract NMJ degeneration.

In recent years, mesenchymal stem cells (MSCs), including amniotic fluid stem cells (AFSCs), have been proposed as a potential therapy for human tissue repair and regeneration, given the encouraging evidence in different experimental neuromuscular disease models [14,15]. Moreover, recent studies demonstrated the potential of MSCs, isolated from different tissues (adipose, umbilical cord, and bone marrow), to induce muscle regeneration [16,17,18]. Human amniotic fluid stem cells (hAFSCs) have different advantages, such as their minimal ethical concerns and being easy to obtain from leftover discarded samples of routine prenatal screening amniocentesis (II trimester of gestation). Among the scientific community, it has become increasingly accepted that the therapeutic potential of these cells can be at least in part attributable to bioactive molecules secreted into extracellular vesicles (EVs). Furthermore, EVs have the advantage of being a cell-free therapy candidate, reducing the risks associated with live cell transplantation. In a recent work, Villa et al. demonstrated the human AFSC pro-survival effect on damaged cardiomyocytes, counteracting apoptosis and mitochondrial impairment [19]. Additionally, the neurogenic potential of these cells has been demonstrated by the presence—although in low amounts—of the neural growth factor BDNF in their EVs cargo, suggesting a neurotrophic activity promoting neuronal survival and neurodevelopmental processes [20]. The beneficial potential of MSC transplantation in amyotrophic lateral sclerosis (ALS) mice has been demonstrated by several studies that have shown a reduction in disease phenotype and progression, but above all else, a partial recovery of motor functions [21,22,23].

It is well known that NMJs become vulnerable in degenerative diseases [24], however evidence on the efficacy of MSC-EVs on NMJ complexes is still lacking. Based on that, the present study aims to explore the paracrine antioxidant and neuroprotective effects of human AFSC-EVs against NMJ perturbations in age-related muscle degeneration. The use of innovative commercially available microfluidic devices allowed us to set up an in vitro model of muscle atrophy induced by glucocorticoid supplementation.

## 2. Results

### 2.1. Effect of Human AFSC-EVs on In Vitro Model of Muscle Atrophy Induced by DEXAMETHASONE

**Figure 1:**
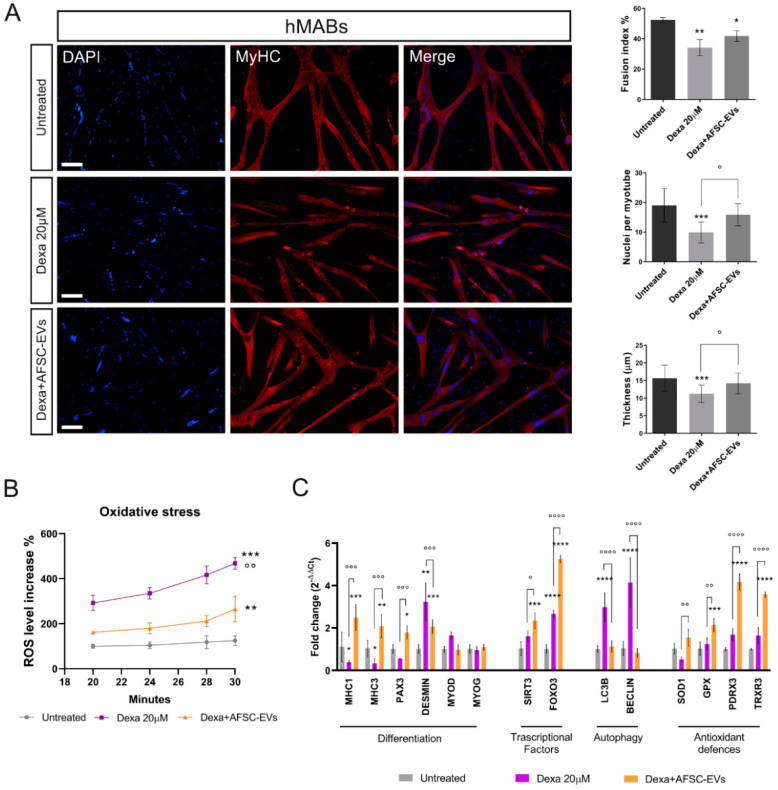
Effect of human AFSC-EV supplementation on in vitro model of hMAB-myotube atrophy. (**A**) Representative images of hMAB-derived myotubes, treated or not with Dexamethasone and AFSC-EVs, stained with myosin heavy chain (MyHC) (red) and DAPI (blue) for nuclei. Scale bars: 50 µm. Graphs relative to analysis of fusion index%, nuclei per myotube, and myotube thickness. Graph data are the mean ± SD (3 biological replicates, 4 fields each replicate). °^,^* *p* value < 0.05, ** *p* value < 0.01, *** *p* value < 0.001. (**B**) Graph showing ROS content in myotubes, pre-treated or not with AFSC-EVs for 24 h, measured from 20 to 30 min after Dexamethasone exposure using DCFH-DA probe. Data shown are the mean ± SD (*n* = 5). Untreated vs. Dexa 20 µM: *** *p* value < 0.001, Untreated vs. Dexa+AFSC-EVs: ** *p* value < 0.01 Dexa 20 µM vs. Dexa+AFSC-EVs: °° *p* value < 0.01. (**C**) Gene expression comparisons among differentiated hMABs (Untreated), Dexa 20 µM, and Dexa+AFSC-EVs. Data shown are the mean ± SD (*n* = 3). °^,^* *p* value < 0.05, **^,^°° *p* value < 0.01, ***^,^°°° *p* value < 0.001, ****^,^°°°° *p* value < 0.0001.

Muscle atrophy was induced in vitro by myotube exposure to 20µM Dexamethasone for 24 h. Preliminary studies have identified this non-cytotoxic concentration (Supplementary Figure S1A,B) as the one able to induce an atrophic phenotype (Supplementary Figure S1C) in hMAB-myotubes. The analysis of immunofluorescence (IF) images of myotubes—stained with myosin heavy chain (MyHC), a typical marker for mature muscle differentiation—showed a reduction in number of nuclei per myotube, and above all, myotube thickness—the main sign of muscle atrophy—after Dexa treatments, while all these typical differentiation indexes were restored by AFSC-EV treatment, although the fusion index was not fully restored (Figure 1A). Moreover, we did not observe significant alterations in the total nuclei number among the different conditions.

In addition, an increased number of MyHC-negative cell nuclei compared to the control one in AFSC-EV-treated samples suggests possible improved preservation of stemness.

Increasing evidence links oxidative stress and reactive oxygen species (ROS) to muscle atrophy [25,26]. Therefore, we decided to investigate the ROS content alteration during the early phase of atrophy induction in hMABs (Figure 1B) by DCFH-DA probe. This analysis showed a significant increase in oxidative stress level, prevented by AFSC-EV exposure.

Moreover, gene expression analysis confirmed the ability of AFSC-EV treatment in restoring the morphological impairment in our in vitro atrophy model (Figure 1C). Indeed, several muscle-specific genes, such as MyHC1, MyHC3, Pax3, and desmin, redox-sensitive signal pathway genes including the forkhead box class O 3 (FOXO3), main regulator of oxidative stress defenses [27], and autophagy-related genes (LC3β and beclin-1) were dysregulated with Dexa treatment. Notably, EV exposure restored the levels of those genes and the increased expression of both FOXO3 and SIRT1 was accompanied by an upregulation of SOD1, GPX, PDRX3, and TrxR3 antioxidant genes.

### 2.2. Effect of Dexamethasone on Mature iPSC-MNs

To generate in vitro functional and morphological mature motor neurons (MNs), we used human-iPSCs differentiated via the 28-day differentiation protocol already published by Guo et al. [28]. The differentiation success was confirmed by gene expression analyses that showed, at day 28, the upregulation of typical pan-neuronal (MAP2 and β-tubulinIII) but also of specific motor neuron (HB9 and Islet-1) markers, and the downregulation of pluripotency markers (NANOG, SOX2), compared to day 10 of differentiation (Supplementary Figure S2A). Additionally, IF images confirmed the positivity of differentiated MNs for synaptophysin (SYPH) and Islet-1 (Isl-1) (Supplementary Figure S2B). Moreover, to be sure that the effect on MNs in co-culture was mediated only by myotubes, we demonstrated that the 20 µM Dexa treatment (24 h) has no effect on motor neuron morphology and differentiation potential (Supplementary Figure S3A), nor does it have a significant impact on the expression of differentiation, redox, and apoptosis marker genes (Supplementary Figure S3B), as demonstrated by immunofluorescence and RT-qPCR analyses.

### 2.3. Distribution of Myotubes and Neurites into Microfluidic Devices—Muscle Compartment and NMJ Formation

**Figure 2:**
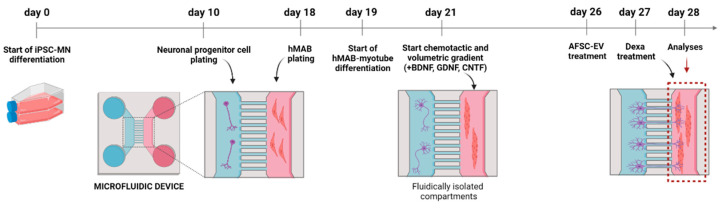
Schematic overview of microfluidic device structure and treatment. Created with BioRender.com (Accessed on 23 February 2023).

To understand the consequences of muscle atrophy on NMJ integrity, we set up an in vitro model of a motor neuron-myotube co-culture using microfluidic devices (Figure 2).

**Figure 3:**
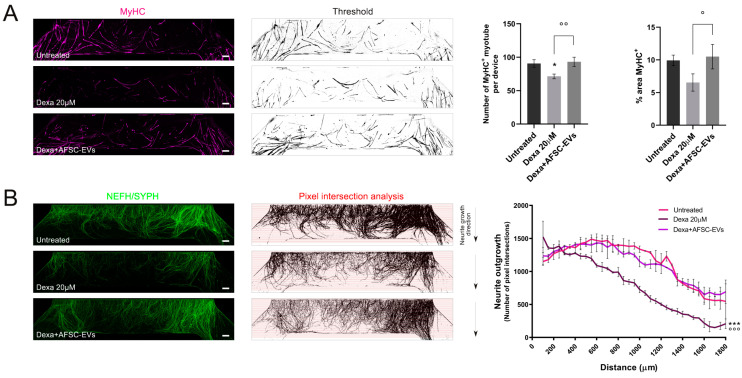
Neurite and hMAB-derived myotube density inside the muscle compartment of microfluidic devices. (**A**) Representative tile scan confocal overviews and quantification graphs of hMAB-derived myotubes (MyHC) into the muscle compartment after Dexa and AFSC-EV treatments. Scale bars: 400 µm. Graph data are the mean ± SD (*n* = 3). °^,^* *p* value < 0.05, °° *p* value < 0.01 (**B**) Representative tile scan confocal overviews and quantification graphs of MN-neurite density (NEFH/SYPH) into the myotube compartment after Dexa and AFSC-EV treatments. Masks of tile scan show intersection lines (red) at every 50 µm starting from the microgroove exit. Arrows: neurite growth direction from the exit of microgrooves. Scale bars: 400 µm. Graph represents quantification of pixel intersection for each intersection line. Graph data are the mean ± SD (*n* = 3). Untreated vs. Dexa 20 µM: *** *p* value < 0.001, Dexa 20 µM vs. Dexa+AFSC-EVs: °°° *p* value < 0.001.

Then, to study the potential of AFSC-EVs in counteracting muscle atrophy injuries, we only treated the muscle compartment with Dexamethasone and examined the modification in myotube and neurite distribution. Dexa treatment reduced the number of MyHC-positive myotubes, while the exposure to EVs recovered the myotube presence (Figure 3A). In parallel, the analysis of neurite density into the muscle compartment was carried out. Interestingly, AFSC-EV exposure was able to restore the neurite density affected during muscle atrophy induction (Figure 3B).

**Figure 4:**
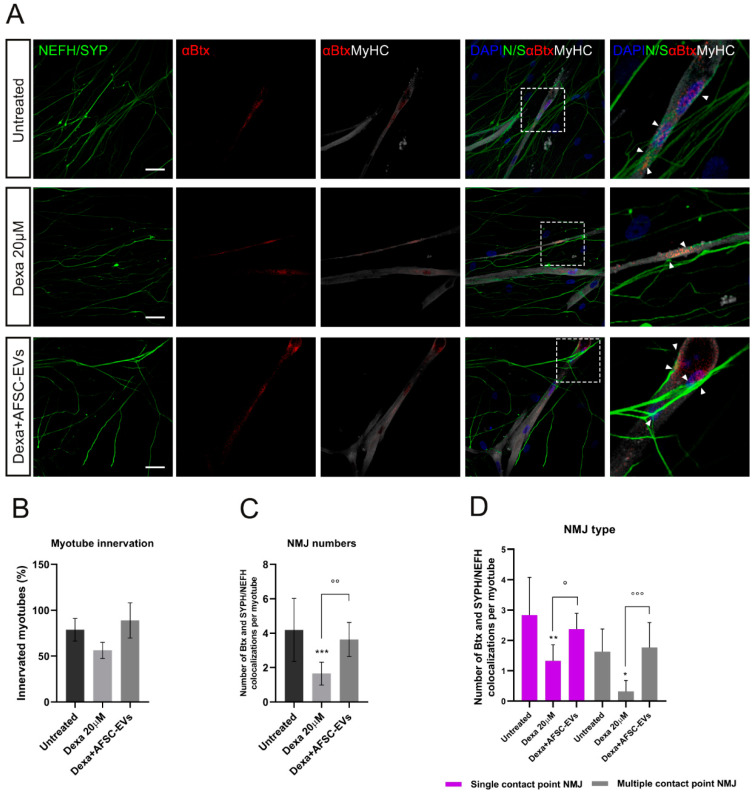
NMJ morphology alteration in vitro model of muscle atrophy and AFSC-EVs therapeutic potential. (**A**) Representative confocal images of NMJs with Dexa and AFSC-EV treatments. NMJs (arrow) are identified as co-localizations between pre-synaptic markers NEFH/SYPH and AChR marker αBtx on MyHC-positive myotubes. Insets: magnification of NMJs. Scale bars: 25 µm. (**B**) Percentage of innervated myotubes. Graph data are the mean ± SD (*n* = 3). (**C**) Number of αBtx and SYPH/NEFH co-localization per myotube. Graph data are the mean ± SD (*n* = 3). °° *p* value < 0.01, *** *p* value < 0.001. (**D**) Quantification of NMJ morphology: single and multiple contact points. Graph data are the mean ± SD (*n* = 3). °^,^* *p* value < 0.05, ** *p* value < 0.01, °°° *p* value < 0.001.

These results led us to study the possible consequences of these impairments on NMJ formation. NMJs were identified as co-localizations between αBtx-positive AChRs and NEFH/SYPH-positive neurites on myotubes (Figure 4A). While the percentage of innervated myotubes was not significantly reduced upon Dexa treatment (Figure 4B), a reduction in NMJ numbers per myotube was observed (Figure 4C) and human AFSC-EVs were effective in counteracting this affection. In addition, NMJs could be distinguished for their morphology as single-contact-point NMJs—less mature—when a neurite touches a AChR cluster one time, or multiple-contact-point NMJs—characteristic of a more mature development state of co-culture—when neurites will fan out and engage with the AChR cluster over a larger surface [29]. Based on this, we observed a reduction in both of these types of interaction in the Dexa-induced atrophy model, while it was prevented by EV pre-exposure (Figure 4D).

### 2.4. Functionality of NMJs after MN-Stimulation in an In Vitro Atrophy Model

**Figure 5:**
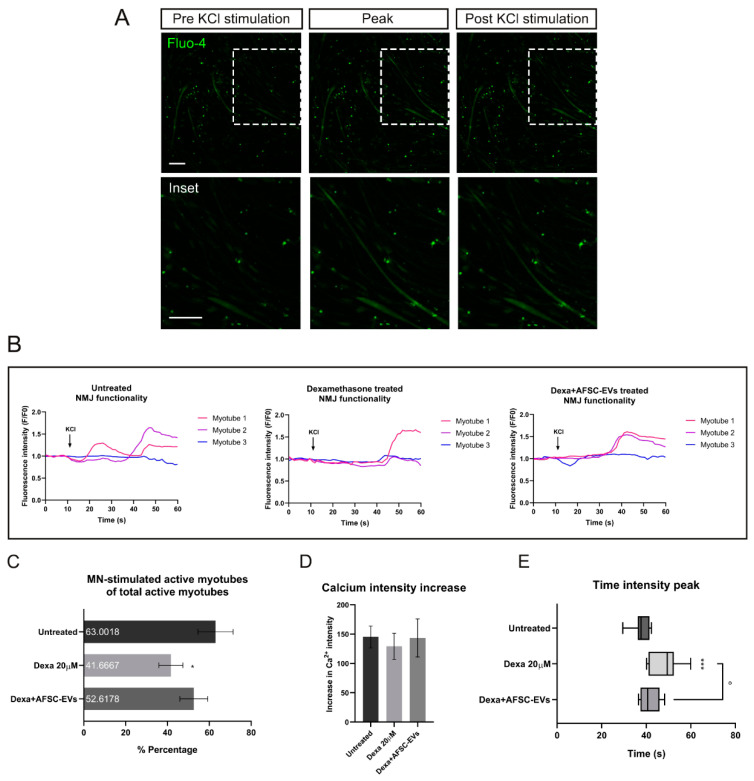
NMJ functionality after Dexamethasone and human AFSC-EV exposure. (**A**) Representative images of MAB-myotubes before, during, and after MN-KCl stimulation labeled with Fluo-4. Scale bars: 200 µm. (**B**) Representative Ca^2+^ influx curves into MAB-myotubes after MN-KCl stimulation (arrow). (**C**) Percentage of active MAB-myotubes after MN-stimulation. Graph data are the mean ± SD (biological replicates = 3, analyzed myotubes: Untreated = 19, Dexa 20 µM = 14, Dexa+AFSC-EVs = 20). * *p* value < 0.05 (untreated vs. Dexa 20 µM). (**D**) Ca^2+^ influx intensity after Dexamethasone and AFSC-EV treatment. Graph data are the mean ± SD (biological replicates = 3, analyzed myotubes: Untreated = 19, Dexa 20 µM = 14, Dexa+AFSC-EVs = 20). (**E**) Graph comparing peak onset times with or without Dexamethasone and AFSC-EV treatment. Graph data are the mean ± SD (biological replicates = 3, analyzed myotubes: Untreated = 19, Dexa 20 µM = 14, Dexa+AFSC-EVs = 20). ° *p* value < 0.05 (Dexa 20 µM vs. Dexa+AFSC-EVs), *** *p* value < 0.001 (untreated vs. Dexa 20 µM).

In order to investigate the functional consequences of morphological alteration in this muscle atrophy model and the therapeutic potential of AFSC-EVs, live-cell calcium imaging was performed (Figure 5A,B). As shown in Figure 5C, Dexa treatments significantly reduced the percentage of motor neuron-stimulated active myotubes, compared to untreated ones, while EV exposure prevented the Dexa-mediated impairment compared to the control. This result brought us to investigate the intracellular calcium transient waves. While significant modifications of the cellular calcium intensity peak were not observed (Figure 5D), this analysis showed a delay of Ca^2+^ peak onset after Dexa treatment, indicating an alteration in myotube functionality. On the other hand, AFSC-EVs were able to reduce this delay in myotube functionality (Figure 5E).

### 2.5. NMJ Oxidative Stress Modulation by Human AFSC-EVs during Atrophy

**Figure 6:**
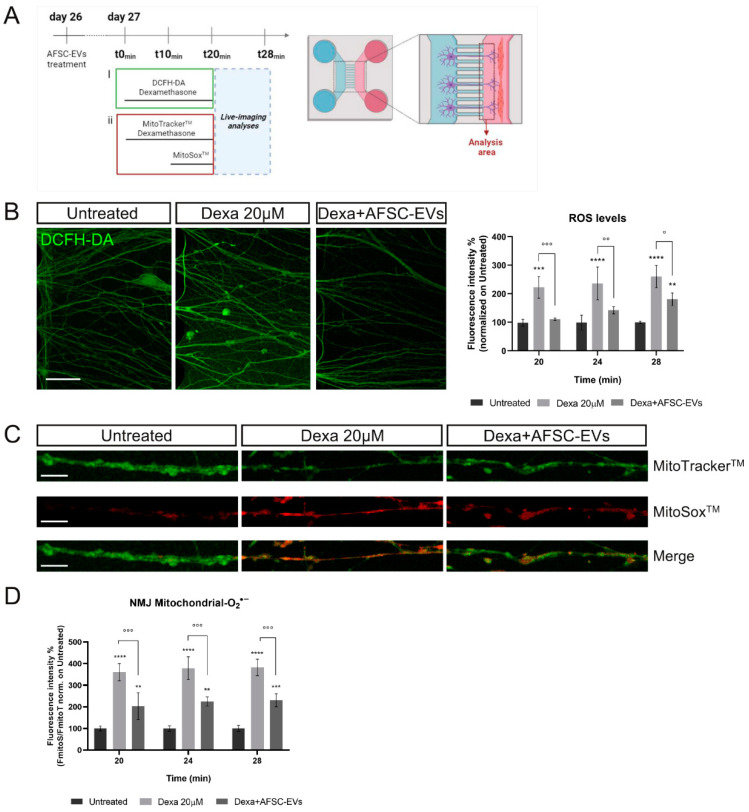
Effect of human AFSC-EVs exposure on MN-intracellular ROS and mitochondrial superoxide content in an in vitro co-culture system of muscle atrophy. (**A**) Schematic overview of time-treatment for ROS and mitochondrial O_2_^•−^ analyses. Created with BioRender.com. (**B**) Representative confocal images of MN-neurites stained with DCFH-DA fluorescent probe (green) into MAB-myotube compartment after 24 min of Dexamethasone exposure in the presence or absence of AFSC-EVs. Scale bar: 50 µm. Graph shows ROS neurite content after 20, 24, and 28 min of Dexamethasone exposure in the presence or absence of AFSC-EVs. Graph data are the mean ± SD (*n* = 3). ° *p* value < 0.05, **^,^°° *p* value < 0.01, ***^,^°°° *p* value < 0.001, **** *p* value < 0.0001. (**C**) Representative confocal images of MN-neurites stained with MitoTracker^TM^ (green) and MitoSox^TM^ (red) after 24 min of Dexamethasone exposure in the presence or absence of AFSC-EVs. Image lengths: 100 µm. Scale bar: 15 µm. (**D**) Fluorescence intensity of MitoSox^TM^/MitoTracker^TM^ recorded after 20, 24, and 28 min of Dexamethasone treatment in the presence or absence of AFSC-EVs. Graph data are the mean ± SD (*n* = 3). °° *p* value < 0.01, ***^,^°°° *p* value < 0.001.

Among many factors, oxidative stress and mitochondrial dysfunction may perform key roles in NMJ decline, muscle strength, and integrity loss [4]. To investigate the oxidative stress alteration in our system, we performed live-imaging assays using fluorescent probes for intracellular ROS and mitochondrial O^2•−^ detection. A schematic overview of experiments is shown in Figure 6A. First, we measured the ROS level variations in neurites after up to 28 min of treatment with Dexa. Dexa exposure increased the ROS content in the neurites that have crossed the microgrooves to contact the myotubes. Notably, EVs protected MN elongations from oxidative stress induced by atrophic muscle cells, in all time points of analysis (Figure 6B).

To investigate the implication of mitochondrial superoxide (O_2_^•−^) in this oxidative context, MitoSox^TM^ live-cell imaging analysis of neurites was performed (Figure 6C). EV pre-treatment was able to reduce mitochondrial O_2_^•−^ levels, increased by Dexa exposure, for every investigated time point (Figure 6D).

## 3. Discussion

In recent years, human AFSCs have been proposed as potential therapeutic approaches for human tissue repair and regeneration, thanks to the encouraging results obtained from different experimental disease models [14,15]. Many of the observed effects can be, at least in part, attributed to the presence of bioactive molecules secreted into extracellular vesicles (EVs), such as antioxidant and anti-inflammatory compounds.

Despite the complexity, the pathogenesis of age-related muscle wasting conditions, such as muscle atrophy, could be linked to a reduction in protein synthesis and/or an enhanced proteolysis, associated with an increase in oxidative stress [30]. In the present study, we aimed to deepen the understanding of the therapeutic potential of EVs, obtained from AFSCs, in rescuing the pathological atrophic phenotypes and detrimental consequences on NMJ integrity induced by Dexa.

Dexa is a synthetic glucocorticoid widely used as a treatment to control different pathological alterations linked to inflammation [31]. Despite its beneficial effects, its abuse can lead to skeletal muscle atrophy, mainly via two pathways: the glucocorticoid receptor (GR)-mediated catabolic processes and the oxidative stress-related pathway [32,33,34]. Given the similar mechanism of muscle damage to age-related atrophy, glucocorticoids are largely used in research for this purpose. FOXO3 plays a crucial role in these catabolic events, regulating both metabolism and oxidative stress defenses. FOXO3 controls the two principal systems of muscle proteolysis: ubiquitin-proteasomal and autophagic/lysosomal pathways. In our in vitro atrophy model, we observed gene expression increase in FOXO3, associated with an overexpression of autophagy-related markers Beclin-1 and LC3β [35]. Moreover, the myotube morphology appeared affected, as shown by the fusion index, nuclei per myotube, and myotube thickness reduction, accompanied by a downregulation of late muscle differentiation markers (myosin heavy chain 1 and 3). Contrarily, the expression of structural muscle protein Desmin appeared increased. Desmin is an intermediate filament fundamental for the maintenance of muscle structure, cellular integrity and size, mitochondrial homeostasis, and proteostasis [36]. Recent studies demonstrated that Desmin gene expression levels increase in different models of heart failure, as a compensatory mechanism for its augmented misfolding and degradation [37,38,39]. Therefore, we assume that a similar self-rescuing response occurred in our atrophy model. Notably, AFSC-EV treatment restored not only the myotube morphology affected by Dexa but also gene expression of all the altered muscle activation/differentiation markers.

Considering the central role of oxidative stress in muscle atrophy progression, and the potential of extracellular vesicles in redox modulation, we investigated it in our Dexa-induced atrophy model. Interestingly, in the presence of EVs, we observed a significant reduction in ROS levels increased by Dexa, accompanied by an upregulation of Sirtuin 3 (Sirt3), FOXO3, and antioxidant genes superoxide dismutase 1 (SOD1), glutathione peroxidase (GPx), peroxiredoxin 3 (PDRX3), and thioredoxin reductase 3 (TrxR3). Sirt3 is a mitochondrial NAD-dependent histone deacetylase (HDAC) principally implicated in stress-adaptive responses by inhibiting mitochondrial oxidative stress. Moreover, the main targets of Sirt3 are FOXO family transcription factors, which once deacetylated increase their transcriptional activity and reduce their degradation via phosphorylation and/or ubiquitination [40]. The FOXO3-activated pathway by Sirtuins upregulates a set of FOXO3a-dependent mitochondrial antioxidant enzymes including superoxide dismutase, thioredoxin, and peroxiredoxin [41]. Additionally, we recently demonstrated that AFSC-EV exposure counteracts oxidative stress in an in vitro model of Alzheimer’s disease and osteoporosis, at least in part by reinforcing the Sirtuin/FOXO antioxidant defenses pathway [42,43]. The results obtained in this work led us to hypothesize a similar mechanism, although it has not been further investigated since our main focus was on NMJ alterations.

Considering the bi-directional communication between nerve and muscle, recent findings have highlighted that skeletal muscle can be a fundamental source of signals for neuron survival, axonal growth, and maintenance of synaptic connection [6]. Based on this, we investigated the muscle impairment consequences on distal axon and the protective potential of vesicles from AFSCs. In order to study the atrophy consequences in a more complex system focusing on NMJs, we used microfluidic devices to set up an in vitro co-culture of human iPSC-derived motor neurons and myotubes with an atrophic phenotype induced by Dexa. The fluidically isolated compartments, where only neurites can growth through microgrooves, not only allows the maintenance of a cell-type-specific microenvironment, but also allows the isolation of subcellular compartments, as distal and proximal parts of the axon, to carry out region-specific analyses [44,45]. Interestingly, the muscle wasting environment induced by Dexa affected the neurite presence in the muscle side and the NMJ maintenance. Furthermore, both types of NMJs—mature multiple contact point and newly formed compensatory single contact point—were impaired due to Dexa treatment. Moreover, AFSC-EVs presence prevented all these neural alterations, probably protecting motor neurons from the detrimental environment created into the synaptic space during atrophy-related muscle wasting. Upon exploring the functional consequences, we noticed a reduction in the number of active myotubes after MN stimulation, compared to the total active myotubes, in the Dexa-atrophy model. Even though we did not observe significant alterations in the calcium influx intensity peak between different conditions during stimulation, the myotube response reactivity to MN stimulation over time was delayed in atrophic conditions. This effect could be reversed by AFSC-EV pre-treatment. Notably, several studies on amyotrophic lateral sclerosis (ALS) models demonstrated that increased oxidative stress and compromised mitochondria, in both muscle and nerve, are among the major contributory factors in affecting presynaptic heath [46,47,48]. In particular, NMJ in vitro exposure to exogenous H_2_O_2_ induces a strong inhibition of spontaneous neurotransmitter release in frog sartorius muscle [49]. In light of this, we propose that the observed alterations in the timing of myotube contraction could be explained as an impairment in the synaptic vesicles’ release by presynaptic terminals affected by an atrophy-related redox imbalance. Nevertheless, we cannot exclude that this may also be due to an impairment in myotube contractile machinery. Furthermore, experiments on mutant SOD1 mouse models demonstrated that oxidative stress originates from distal muscles before the onset of ALS pathology. This suggests that oxidative damage starts at the postsynaptic side of the muscle and propagates to motor neurons’ presynapse and further up to the axon in a retrograde manner towards the neuronal soma, ultimately leading to apoptosis of the entire cell [50,51,52]. To evaluate this hypothesis in our model, the oxidative stress and the mitigating effect of EVs in atrophy-related NMJ alterations were investigated. To this purpose, we followed the redox modification of neurites reaching the myotube area, the ones likely creating NMJs. We observed that the treatment with AFSC-EVs reduced not only the increased ROS levels but also the mitochondrial superoxide (O_2_^•−^) overproduction in neurites, associated with muscle atrophy induction. Antioxidant proteins carried by AFSC-EVs, including SOD1, could have a direct effect on ROS scavenging, re-equilibrating the redox balance affected in NMJs. Importantly, increased local activities of ROS are linked to a reduction in motor neuron and NMJ integrity and efficiency in muscle contraction, and the permanence of this oxidative stimuli leads to a permanent disruption in their structure and functionality [53,54]. The analysis of our data demonstrated that the re-equilibration of redox balance by AFSC-EVs, together with their immunomodulatory and neurogenic activity, have a protective effect on NMJ and motor neuron damage associated with muscle atrophy.

## 4. Materials and Methods

### 4.1. Human Amniotic Fluid Collection

The human amniotic fluid stem cells (hAFSCs) were obtained from amniotic fluids collected from 3 healthy pregnant women at the 16th week of gestation who underwent amniocentesis per maternal request (not foetal anomalies) at the Unit of Obstetrics and Gynecology, Policlinico Hospital of Modena (Modena, Italy). The amniocentesis was performed under continuous ultrasound guidance, in a sterile field, with a 23-Gauge needle. The risk related to the procedure and the purpose of the study were explained to all patients before the invasive procedure, and the ob-gyn specialist collected a signed consent form before starting the exam (protocol 360/2017, dated 15 December 2017 and approved by Area Vasta Emilia Nord). For this study, unused (supernumerary) flasks of AF cells cultured in the Laboratory of Genetics of the TEST Lab (Modena, Italy) for 2 weeks were used.

### 4.2. Human Amniotic Fluid Stem Cell Isolation and Culture

hAFSCs were isolated as previously described [55]. Briefly, human amniocentesis cultures were harvested by trypsinization and subjected to c-kit immunoselection by MACS technology (Miltenyi Biotech, Germany). hAFSCs were subcultured routinely at 1:3 dilution and not allowed to grow beyond 70% confluency. hAFSCs were grown in αMEM culture medium (Corning, Manassas, VA, USA) supplemented with 20% fetal bovine serum (FBS) (Gibco, Waltham, MA, USA), 2 mM L-glutamine, 100 U/mL penicillin, and 100 µg/mL streptomycin (all reagents from EuroClone Spa, Milano, Italy).

### 4.3. Extracellular Vesicles Isolation

hAFSCs were grown in 75cm^2^ flasks until sub-confluence (around 10^6^ cells). Then, cells were maintained in FBS-free culture medium (10 mL) for 4 days, to avoid contamination by EVs from the FBS solution. The collected conditioned medium (CM) was centrifuged at 300× *g* for 10 min at 4 °C to eliminate cellular debris, and then concentrated up to 2 mL by using centrifugal filter units with a 3K cut-off (Merk Millipore, Burlington, MA, USA) [54]. The concentrated CM was again centrifuged at 10,000× *g* for 30 min at 4 °C and then, the supernatant was ultracentrifuged in polypropylene ultracentrifuge tubes (13.5 mL, Beckman Coulter) at 100,000× *g* for 90 min at 4 °C in a Beckman Coulter Optima L-90 K centrifuge (SW-41 rotor); the supernatants were discarded and the pellets were resuspended in 13 mL DPBS (Corning, Manassas, VA, USA) and ultracentrifuged again (100,000× *g*, 90 min at 4 °C) [56]. The pellet was resuspended in 100 µL of DPBS for subsequent analyses and treatments. Size distribution and concentration of EVs were analyzed, after a 1:100 dilution, by a NanoSight particle tracker from NanoSight Ltd. (Malvern Panalytical, Worcestershire, UK).

### 4.4. Derivation, Maintenance, Differentiation, and Treatment of Human Mesoangioblasts

Human Mesoangioblasts (hMABs) were isolated as previously described [57,58]. hMABs were cultured on collagen from calf skin-coated flasks in IMDM growth medium (Sigma, Milan, Italy) supplemented with 1% sodium pyruvate, 1% non-essential amino acids, 1% L-glutamine, 1% insulin transferrin selenium (all reagents from EuroClone Spa, Milano, Italy), 5 ng/mL recombinant human basic fibroblast growth factor (bFGF) (PeproTech, Rocky Hill, NJ, USA). Medium was changed every 3 days. Since physical contact between hMABs initiates fusion and reduces the myogenic potential, cells were trypsinized before 70% confluency [59]. To induce myotube differentiation, confluent hMABs were exposed for 1 week to a differentiation medium composed of 1:1 DMEM/F12 (Life Technologies, Thermo Fisher Scientific, Waltham, MA, USA) supplemented with 2% horse serum (Thermo Fisher Scientific, Waltham, MA, USA) and 1% sodium pyruvate (EuroClone Spa, Milano, Italy) on collagen from calf skin-coated supports. In order to induce muscle atrophy, after 7 days myotubes, were treated with 20 µM Dexamethasone (Dexa) (Sigma Aldrich, St Louis, MO, USA) in differentiation medium for 20 min (for ROS analysis) or 24 h (for all other experiments). AFSC-EVs t (1.3 × 10^8^ particles/cm^2^) were added 24 h before Dexa treatment and maintained for the glucocorticoid treatment time.

### 4.5. Differentiation of iPSCs into Mature Motor Neurons and Treatment

To obtain mature motor neurons (MNs) from iPSCs (Gibco^TM^ Episomal hiPSC Line) (Gibco, Thermo Fisher Scientific, Waltham, MA, USA), the protocol by Dittlau et al. was applied [28,29]. Briefly, iPSCs were harvested using Collagenase type IV (Gibco, Waltham, MA, USA), transferred into Ultra-low attachment multi6-well plates (Corning Manassas, VA, USA) to promote cluster formation and maintained in Neuronal medium (50% DMEM/F12 and 50% Neurobasal Medium (both from Life Technologies, Thermo Fisher Scientific, Waltham, MA, USA) with 2 mM L-glutamine, 100 U/mL penicillin, and 100 µg/mL streptomycin (all reagents from EuroClone Spa, Milano, Italy), 1% N2 supplement, 2% B-27^TM^ without vitamin A, 0.1% β-mercaptoethanol (all reagents from Thermo Fisher Scientific, Waltham, MA, USA), 0.5 µM ascorbic acid (Sigma-Aldrich, Milan, Italy)) supplemented with 5 µM Y-27632 (Merck Millipore, Burlington, MA, USA), 0.2 µM LDN-193189 (Stemgent, Beltsville, MA, USA), 40 µM SB431542, and 3 µM CHIR99021 (both from Tocris Bioscience, Bristol, UK) for 2 days, changing the medium every day. From day 2, Neuronal medium was supplemented with 0.1 µM retinoic acid (Sigma-Aldrich, Milan, Italy), 500 nM smoothened agonist (SAG) (Merck Millipore, Burlington, MA, USA), and from day 7, brain-derived neurotrophic factor (BDNF) and glial cell-derived neurotrophic factor (GDNF) (both from PeproTech, Rocky Hill, NJ, USA) were added. On day 9, 20 µM DAPT (Tocris Bioscience, Bristol, UK) was added. On day 10, single cells were obtained from floating clusters by 0.05% trypsin (Gibco, Waltham, MA, USA) treatment, and seeded onto poly-L-ornithine- (PLO) and laminin- (both from Sigma, St Louis, MO, USA) coated plates. Single cell neural progenitor cells (NPCs) were maintained in culture until experiments in Neuronal medium supplemented with BDNF, GDNF, and CNTF (ciliary neurotrophic factor) were conducted, and the medium was refreshed every 2 days. To test the effect of Dexa on mature motor neurons, at day 27 of differentiation, Dexa treatment was applied at a concentration of 20 µM for 24 h.

### 4.6. Preparation of Microfluidic Devices

Microfluidic devices (Xona^TM^ Microfluidics, Temecula, CA, USA; Cat N° XC150) were sterilized in 90% ethanol and left to air-dry in a sterile laminar flow hood. Devices were placed individually in 10 cm petri dishes for easy handling. Before cell seeding, devices were coated using 100 µg/mL poly-L-ornithine (PLO) in DPBS for 3 h and then 20 µg/mL laminin (both from Sigma, St Louis, MO, USA) in Neurobasal medium (Life Technologies, Thermo Fisher Scientific, Waltham, MA, USA) overnight. All coated materials were incubated at 37 °C, 5% CO_2_, and a volume difference of 100 µL between two sides was applied to allow the coating to pass through the microgrooves (maximum capacity for each device well: 200 µL). The day after, devices were carefully washed once with DPBS before neural cell plating.

### 4.7. Co-Culturing Myotubes and MNs in Microfluidic Devices and Treatments

Myotubes and MNs were co-cultured into Xona^TM^ microfluidic devices according to a previously described protocol [59]. Briefly, day 10 MN-precursor cells were seeded into one side of devices at a seeding density of 1.25 × 10^5^ cells/well (total 2.5 × 10^5^ cells/side) and maintained in day-specific differentiation medium according to the differentiation protocol. After 1 week (day 17), hMABs were seeded into the opposite compartment (10^5^ cells/well, total 2 × 10^5^ cells/side), and the day after (day 18), myotube differentiation was started (differentiation medium described in section “Derivation, Maintenance, Differentiation, and Treatment of Human Mesoangioblasts”). On day 21, a chemotactic and volumetric gradient was established: neuronal compartments received 100 µL/well of neuronal medium without neurotrophic factors, while myotube compartments received 200 µL/well neuronal medium supplemented with 30 ng/mL BDNF, GDNF, CTNF (all reagents from PeproTech, Rocky Hill, NJ, USA), 20 µg/mL laminin (Sigma, St Louis, MO, USA), and 0.01 µg/mL recombinant human agrin protein (R&D Systems, Minneapolis, USA). The gradient and laminin/agrin treatment were maintained until the end of the co-culture period. At day 26, AFSC-EVs were added to both compartments in day 21 medium (4.16 × 10^7^ particles/well), and after 24 h, only the myotube compartment was also treated with 20 µM Dexa for 20 min for oxidative stress analysis or 24 h for all other analyses.

### 4.8. RNA Isolation and Quantitative Real-Time PCR

For the quantitative Reverse Transcription Polymerase Chain Reaction (RT-qPCR) assay, the Purelink^®^ RNA mini kit (Thermo Fisher Scientific, Waltham, MA, USA) was used to isolate total RNA, and RNA samples were purified by a TurboTM DNA-free kit (Thermo Fisher Scientific, Waltham, MA, USA) following manufacturers’ instructions. First, 1 µg of RNA was reverse-transcribed using the Superscript III Reverse Transcriptase First-Strand Synthesis SuperMix (Thermo Fisher Scientific, Waltham, MA, USA), according to the manufacturers’ protocol. Then, Platinum SYBR Green QPCR SuperMix-UDG (Thermo Fisher Scientific, Waltham, MA, USA) was used to dilute cDNA (1:5). The RT-qPCR was performed by a Viia7 384-plate reader (Thermo Fisher Scientific, Waltham, MA, USA) [60]. Oligonucleotide primer forward/reverse sequences are listed in Table 1.

### 4.9. Immunofluorescence Confocal Microscopy and Image Analysis

For immunofluorescence analysis, cells—seeded on coverslips or into microfluidic devices—were processed and confocal imaging was performed using a Nikon A1 confocal laser scanning microscope, as previously described [43,59]. Primary antibodies to detect neurofilament heavy chain (NEFH) (Abcam, Cambridge, UK), synaptophysin (SYPH) (Cell Signaling Technology, Lieden, Netherlands), Islet-1 (Isl-1) (Millipore, Burlington, MA, USA), β-tubulinIII (βtubIII) (Cell Signaling Technology, Lieden, Netherlands), and myosin heavy chain (MyHC) (In-house, SCIL, dil. 1:20) were used following the datasheet-recommended dilutions. α-Bungarotoxin-tetramethylrhodamine (Sigma-Aldrich, MO, USA) was incubated with secondary antibodies according to the manufacturers’ protocol. Alexa secondary antibodies (Thermo Fisher Scientific, Waltham, MA, USA) were used at a 1:200 dilution. To obtain three-dimensional projections, the confocal serial sections were processed with ImageJ software [61], while image rendering was performed with Adobe Photoshop software [62]. For myotube fusion index, nuclei per myotube, myotube thickness analyses, and NMJ quantifications, MyHC-positive cells containing multiple nuclei were selected as myotubes. Fusion index percentage was calculated as a ratio percentage between the number of nuclei inside myotubes and total nuclei. Myotube thickness was measured using ImageJ software. For NMJ quantification into microfluidic devices, 40× magnification images of MyHC-positive myotubes were collected using an inverted confocal microscope. The number of co-localizations between NEFH/SYPH and α-bungarotoxin (αBtx) (Sigma, St Louis, MO, USA), for Acetylcholine Receptor (AChR) identification, was counted manually through each z-stack, and the number of co-localizations was normalized to the number of myotubes present in the z-stack. NMJ morphology, single or multiple contact point, was analyzed looking at neurite interactions with AChR clusters, as previously described by Dittlau et al., 2021 [29]. Briefly, NMJs were identified as a single contact point when a neurite touched a AChR cluster once, while a multiple contact point was defined as a neurite fanning out and engaging with the AChR cluster over a larger surface.

### 4.10. Neurite Density-Outgrowth Quantification

Neurite density-outgrowth quantification was performed as previously described by Dittlau et al. 2021 [29]. Briefly, tile scan images of NEFH fluorescence were taken using an inverted Leica SP8 DM18 confocal microscope and neurites were isolated using Ilastik 1.3.3post1 Pixel Classification software. A custom ImageJ 1.52p software linear School analysis script was used to quantify the total number of pixels that intersect an intersection line (distance between lines: 50 µm). The measuring was started at a 100 µm distance from the microgrooves due to the high neurite density at the exit of microgrooves.

### 4.11. Calcium Fluorescent Imaging

After AFSC-EV and/or Dexamethasone treatments, the myotube compartment was incubated with 5 µM Fluo-4 AM (Thermo Fisher Scientific, Waltham, MA, USA) for 25 min in the dark (5% CO_2_, 37 °C). MNs were stimulated with 50 mM KCl and Fluo-4 fluorescence was recorded in the myotube compartment (1 picture/second for a total of 60 s, 10× magnification). Calcium transients were recorded after KCl stimulations in two different fields for each replicate. The fluidic isolation of the compartments in the microfluidic devices ensured no direct contact between the high KCl solution and the myotubes. To verify the myotube functionality, a positive test was performed by direct stimulation of myotubes with 50 mM KCl in the myotube compartment. The percentage of MN-stimulated active myotubes was calculated as a ratio between MN-stimulated active myotubes and total active myotubes. Recordings were acquired and analyzed using a Nikon A1R confocal microscope and NIS-Elements AR 4.30.02 software. Calcium waves were calculated as a ratio between the myotube fluorescence at each analyzed point and the fluorescence mean during the first 5 s of recording. Time intensity peak was calculated considering the time of peak starting onset.

### 4.12. Viability Assay

hMABs were seeded and differentiated into 96-well plates (5 × 10^5^ cell/well) with 5 replicates for each condition. After 7 days, myotubes were treated with 1, 10, 20, or 40 µM Dexamethasone (Dexa) (Sigma Aldrich, St Louis, MO, USA) in differentiation medium for 24 h. At the end of the treatments, 0.5 mg/mL MTT was added and incubated for 3 h at 37 °C. After incubation, the medium was removed, and acidified isopropanol was added to solubilize the formazan salts. The adsorbance was measured at 570 nm using a microplate spectrophotometer (Appliskan, Thermo-Fisher Scientific, Vantaa, Finland).

### 4.13. ROS Detection

To evaluate the intracellular ROS levels, a dichlorodihydrofluorescein diacetate (DCFH-DA) assay was performed as previously described [63]. For myotube oxidative stress investigation, hMABs were seeded and differentiated into 96-well plates (5 × 10^5^ cell/well) with 5 replicates for each condition, while for co-culture oxidative stress analysis, myotubes and MNs were cultured in microfluidic devices as described above. Culture medium was removed from each well and 5 µM DCFH-DA was incubated in PBS with 1 gr/L of glucose for 20 min at 37 °C and 5% CO_2_. Dexa 20 µM treatment was only added to myotubes with the probe in the meantime and maintained for 20 min. In the 96-well plates, the probe solution was replaced with PBS/glucose and the fluorescence was read at 485 nm (excitation) and 535 nm (emission) using the multiwell reader Appliskan (Thermo Fisher Scientific, Waltham, MA, USA). Cellular autofluorescence was subtracted as a background using the values of the wells not incubated with the probe. For devices, after 20 min of Dexa treatment, the probe solution was replaced with PBS/glucose buffer and the neurite fluorescence was recorded for 8 min into the muscle compartment with a Nikon A1 confocal laser scanning microscope equipped with a live-cell imaging system. Live images were taken between microgroove exits and myotubes in order to select the neurites most likely to have contacted myotubes and to avoid myotube fluorescence noise.

### 4.14. Mitochondrial Oxidative Stress Analysis

Confocal images were obtained using a Nikon A1 confocal laser scanning microscope equipped with a live-cell imaging system. During live imaging, cells were maintained in a PBS-glucose (1g/L) buffer at 37 °C, 5% CO_2_. All acquisition settings, including detector sensitivity and camera exposure time, were maintained constant during recording. To avoid photobleaching and to reduce cell stress, laser power was set to minimum. To identify mitochondria, at day 27—after 24 h of AFSC-EVs exposure—both microfluidic device compartments were washed once with PBS/glucose buffer and then incubated with 100 nm MitoTracker^TM^ Green FM probe (Invitrogen, Waltham, MA, USA) in PBS/glucose buffer, and, in only the myotube compartment, with 20 µM Dexa for 20 min at 37 °C, 5% CO_2_. After the first 10 min, 5 µM MitoSox^TM^ Red was added to both compartments to identify mitochondrial superoxide production. After the incubation time, microfluidic devices were gently washed 3 times and maintained in PBS/glucose during live imaging analysis. MitoSox^TM^ and MitoTracker^TM^ fluorescence was recorded in myotube compartments next to the microgrooves exit (20× magnification, with 10 s interval for a duration of 8 min). MitoSox^TM^ signal was normalized on MitoTracker^TM^ for each time point.

### 4.15. Statistics

All the experiments were performed with 3 biological replicates. For quantitative comparisons, the values were reported as the mean ± SD based on a triplicate analysis for each sample. One-way ANOVA with a Bonferroni post hoc test or a Student’s *t*-test were applied to test the significance of the observed differences amongst the study groups. Statistical significance was considered as a *p*-value < 0.05. Statistical analysis and plot layout were obtained by using GraphPad Prism^®^ release 8.0 software.

## 5. Conclusions

In this study, we investigated the protective effect of AFSC-EV treatment upon Dexa-induced muscle atrophy and its consequences on the presynaptic part of NMJs. We took advantage of the microfluidic human MAB/iPSC-MN co-culture system to study muscle-nerve cross-communication during muscle atrophy. Glucocorticoids exposure confirmed the neurodegeneration induced by muscle atrophy; however, the AFSC-EV administration ameliorated the disease progression, thanks also to their ROS regulation capability. While this study is descriptive in nature, it is providing evidence for beneficial effects of AFSC-EVs on NMJs alterations transmitted by muscle atrophy, and this microfluidic NMJ system can be further explored for small molecule screening and mechanistic follow-up studies.

## Figures and Tables

**Table 1:** List of primers used for gene expression analysis.

Gene	Primer Sequence
h-SIRT2	Fw: CTGCGGAACTTATTCTCCCAGAC Rev: CCACCAAACAGATGACTCTGCG
h-SIRT3	Fw: GCTGGACAGAAGAGATGC Rev: GTGGATGTCTCCTATGTTACC
h-FOXO3	Fw: TCAAGGATAAGGGCGACAGC Rev: GGACCCGCATGAATCGACTA
h-LC3β	Fw: GAGAAGCAGCTTCCTGTTCTGG Rev: GTGTCCGTTCACCAACAGGAAG
h-BECLIN	Fw: CCATGCAGGTGAGCTTCGT Rev: GAATCTGCGAGAGACACCATC
h-MHC1	Fw: GCTGGCTAAGACCGAGGCAAAA Rev: CCTTTCCTCTGCATCAGCCAAG
h-MHC3	Fw: CTGGAGGATGAATGCTCAGAGC Rev: CCCAGAGAGTTCCTCAGTAAGG
h-PAX	Fw: GGGCCTCCTGCTTGTTTAT Rev: CCATCTGGCTGGACTTCAAT
h-DESMIN	Fw: GAAGCTGCTGGAGGGAGAG Rev: ATGGACCTCAGAACCCCTTT
h-SOD1	Fw: GGTGGGCCAAAGGATGAAGAG Rev: CCACAAGCCAAACGACTTCC
h-GPX	Fw: CAGTCGGTGTATGCCTTCTCG Rev: GAGGGACGCCACATTCTCG
h-PDRX3	Fw: ACAGCCGTTGTCAATGGAGAG Rev: ACGTCGTGAAATTCGTTAGCTT
h-TRXR3	Fw: ATGAGGCTGTTAGACCTCTGC Rev: GGCGACAATGGCATTCACATC
h-NANOG	Fw: CTCCAACATCCTGAACCTCAGC Rev: CGTCACACCATTGCTATTCTTCG
h-OCT4	Fw: CCTGAAGCAGAAGAGGATCACC Rev: AAAGCGGCAGATGGTCGTTTGG
h-SOX2	Fw: GCTACAGCATGATGCAGGACCA Rev: TCTGCGAGCTGGTCATGGAGTT
h-SOX17	Fw: ACGCTTTCATGGTGTGGGCTAAG Rev: GTCAGCGCCTTCCACGACTTG
h-NESTIN	Fw: TCAAGATGTCCCTCAGCCTGGA Rev: AAGCTGAGGGAAGTCTTGGAGC
h-OLIG2	Fw: ATGCACGACCTCAACATCGCCA Rev: ACCAGTCGCTTCATCTCCTCCA
h-HB9	Fw: GCCTAAGATGCCCGACTTCAAC Rev: CGCGACAGGTACTTGTTGAGCT
h-ISL1	Fw: GCAGAGTGACATAGATCAGCCTG Rev: GCCTCAATAGGACTGGCTACCA
h-MAP2	Fw: AGGCTGTAGCAGTCCTGAAAGG Rev: CTTCCTCCACTGTGACAGTCTG
h-βTUB3	Fw: TCAGCGTCTACTACAACGAGGC Rev: GCCTGAAGAGATGTCCAAAGGC
h-CASP3	Fw: GGAAGCGAATCAATGGACTCTGG Rev: GCATCGACATCTGTACCAGACC
h-CASP8	Fw: AGAAGAGGGTCATCCTGGGAGA Rev: TCAGGACTTCCTTCAAGGCTGC
h-CASP9	Fw: GTTTGAGGACCTTCGACCAGCT Rev: CAACGTACCAGGAGCCACTCTT
h-GAPDH	FW: 5′-TCAAGAAGGTGGTGAAGCAGG-3′ RV: 5′-ACCAGGAAATGAGCTTGACAAA-3′

## Data Availability

Not applicable.

## Supplementary Materials

The following supporting information can be downloaded at: https://www.mdpi.com/article/10.3390/ijms24054944/s1.
