# Supplementary material for: Human Neuromuscular Junction on a Chip: Impact of Amniotic Fluid Stem Cell Extracellular Vesicles on Muscle Atrophy and NMJ Integrity
Source: Int J Mol Sci. 2023 Mar 3;24(5):4944. doi: 10.3390/ijms24054944 (PMC10003237; doi:10.3390/ijms24054944)
Supplement: Supplementary file 1 [file ijms-24-04944-s001.zip › ijms-2129338-supplementary.pdf]

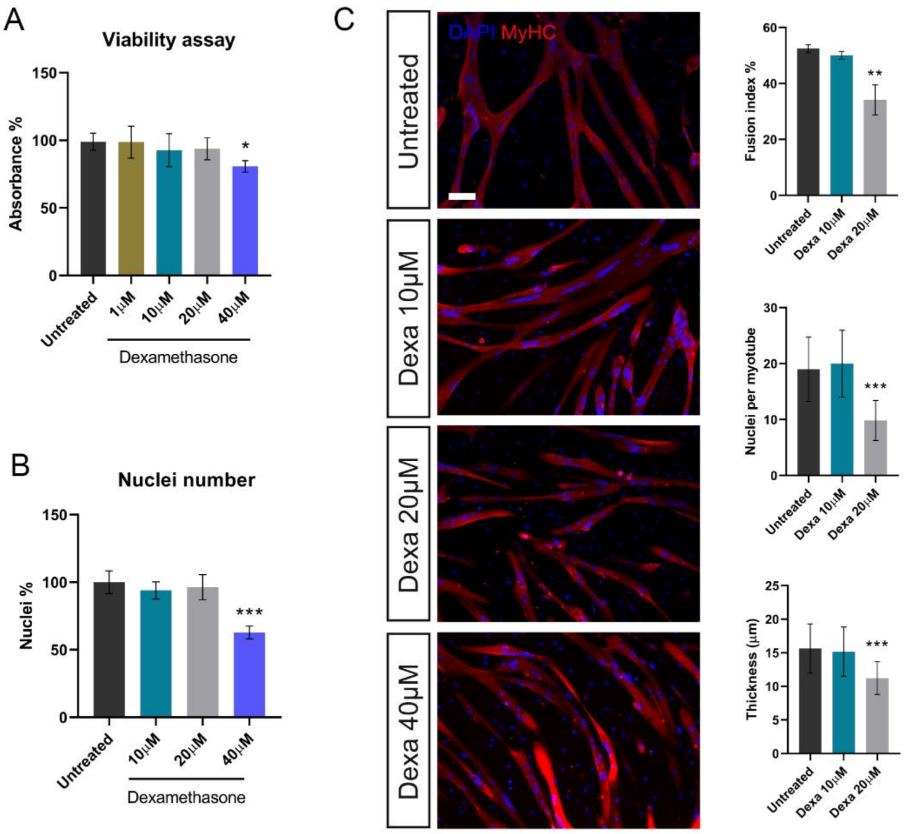


**Figure S1.** Dose dependent effects of Dexamethasone on hMAB-myotubes. (**A**) Graph of MTT assay of hMAB-myotubes exposed to 1, 10, 20, and 40 μM Dexamethasone for 24 hours. Data shown are the mean ± SD (n=5). *$p$ value < 0.05. (**B**) Graph relative to the analysis of total nuclei number variations after 10, 20, and 40 μM Dexamethasone exposure for 24 hours. Data shown are the mean ± SD (biological replicates=3, fields each replicate =4). ***$p$ value < 0.001. (**C**) Representative images of hMAB-derived myotubes, treated or not with 10, 20, and 40 μM Dexamethasone for 24 hours, stained with myosin heavy chain (MyHC) (red) and DAPI (blue) for nuclei. Scale bars: 50μm. Graphs relative to analysis of fusion index%, nuclei per myotube, and myotube thickness after Dexamethasone exposure (20 and 40 μM for 24 hours). Data shown are the mean ± SD (biological replicates=3, fields each replicate =4). **p value < 0.01, ***p value < 0.001.

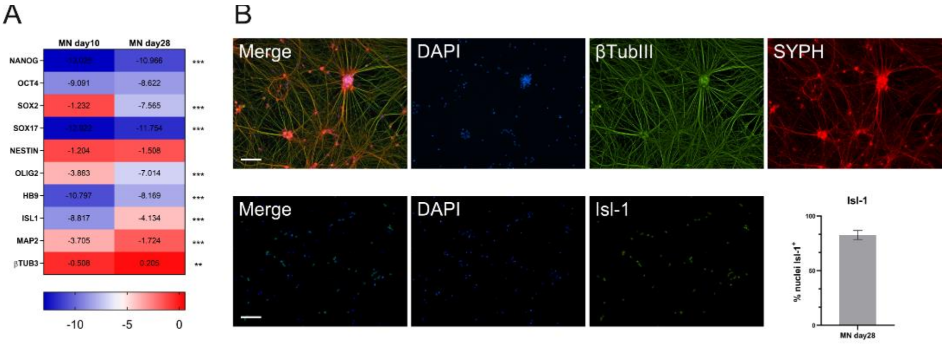


**Figure S2.** Differentiation of mature motor neurons derived from iPSCs. (**A**) Graph showing gene expression comparison between motor neurons at day 10 and 28 of differentiation for pluripotency, pan-neuronal, and motor neuron-specific markers. Data shown are the mean ± SD (n=3). ***p* value < 0.01, ****p* value < 0.001 (**B**) Representative confocal images of MNs at day 28 of differentiation stained with βtubulinIII (βtubIII) (green), synaptophysin (SYPH) (red) – top –, and Islet-1 (Isl-1) (green) – bottom. Nuclei stained with DAPI. Scale bars: 75μm.

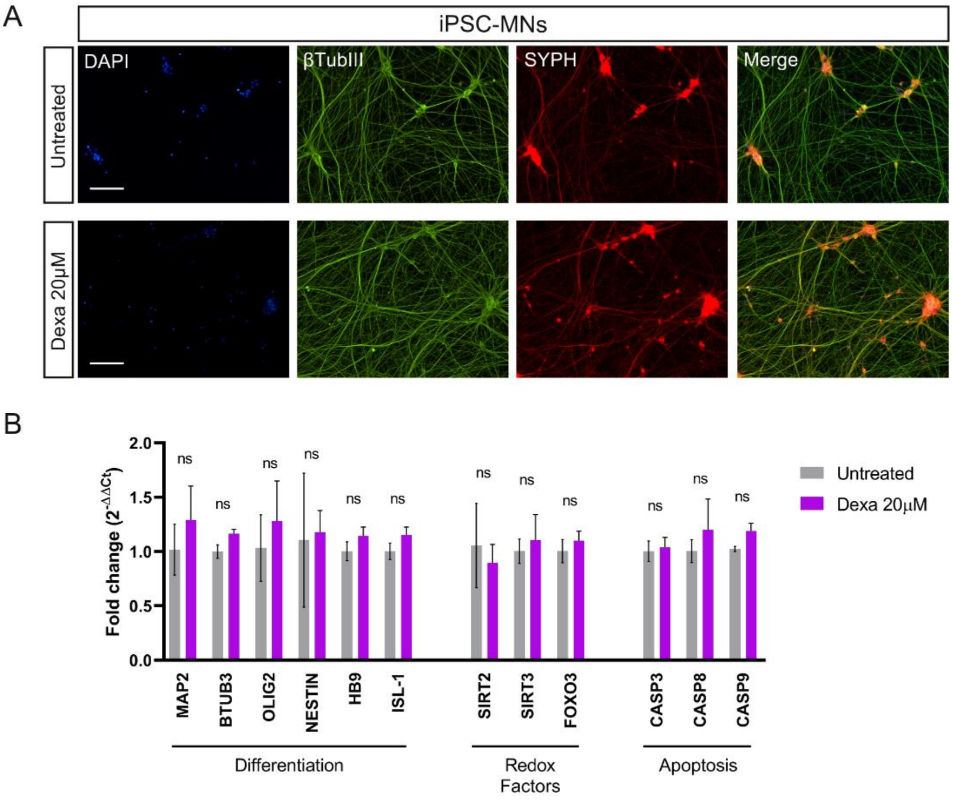


**Figure S3.** Dexamethasone effect on mature human iPSC-derived motor neurons. (**A**) Representative images of iPSC-derived motor neurons (iPSC-MNs) treated or not with 20 μM Dexamethasone (24 h) stained with βtubulinIII (βtubIII) and synaptophysin (SYPH). Nuclei stained with DAPI. Scale bars: 75μm. (**B**) Graph showing gene expression comparison of neuronal differentiation, redox factor, and apoptosis markers of MNs treated or not with Dexamethasone. Data shown are the mean ± SD (n=3).
